# In vitro Cytotoxicity Effects of ^197^Au/PAMAMG4 and ^198^Au/PAMAMG4 Nanocomposites Against MCF7 and 4T1 Breast Cancer Cell Lines

**DOI:** 10.15171/apb.2017.011

**Published:** 2017-04-13

**Authors:** Simin Janitabar-Darzi, Reza Rezaei, Kamal Yavari

**Affiliations:** ^1^Radiopharmaceutical Research and Development Laboratory, Nuclear Science and Technology Research Institute, Tehran, Iran.; ^2^Department of biochemistry, Faculty of Science, Zanjan University, Zanjan, Iran.

**Keywords:** ^198^Au/PAMAMG4, 4T1, MCF7, C2C12, Nanocompsite, In vitro

## Abstract

***Purpose:*** Study on gold based therapeutic agents for cancer cells deracination has become under scrutiny in recent years owing to effective treatments are not available for rapidly progressive cancers. The aim of present study was to examine efficiency of radioactive ^198^Au/PAMAMG4 and non-radioactive ^197^Au/PAMAMG4 nancomposites against 4T1 and MCF7 breast cancer cell lines.

***Methods:*** The PAMAMG4 dendrimer was treated with the gold anions and then, the mixture was chemically reduced by NaBH_4_. Prepared ^197^Au/PAMAMG4 was bombarded by thermal neutrons in the Tehran Research Reactor to ^198^Au/PAMAMG4 be produced. Prepared nanocomposites were characterized by means of FT-IR, 1H NMR, Zeta-potential measurements, TEM and EDX analyses. The radionuclidic purity of the ^198^Au/PAMAMG4 solution was determined using purity germanium (HPGe) spectroscopy and its stability in the presence of human serum was studied. In vitro studies were carried out to compare toxicity of PAMAMG4, ^197^Au/PAMAMG4 and ^198^Au/PAMAMG4 towards 4T1 and MCF7 cancerous cells and C2C12 normal cell lines.

***Results:*** Characterization results exhibited that invitro agents, ^197^Au/PAMAMG4 and ^198^Au/PAMAMG4, were synthesized successfully. Cells viability after 24 h, 48 h, and 72 h incubation, using MTT assay showed that the toxicity of ^198^Au/PAMAMG4 is significantly superior in comparison with ^197^Au/PAMAMG4 and PAMAMG4. Furthermore, the toxicity of ^198^Au/PAMAMG4 was higher on cancerous cells especially in higher level of concentrations after 72 hours (P<0.05).

***Conclusion:*** In the current study, the preparation of ^197^Au/PAMAMG4 and ^198^Au/PAMAMG4 is described and the cytotoxic properties of them against the MCF7, 4T1 cancerous cells and C2C12 normal cells were evaluated using MTT assay.

## Introduction

Gold nanoparticles (NPs) have recently received great technological and scientific interest due to their extensive applications in biology, catalysis, and nanotechnology.^1^ Some polymer-based methods in order to preparation of AuNPs are reported up to now.^2-6^ However, the dendrimer templating procedure for producing gold NPs is known as a powerful method to form organic-inorganic nanocomposites hybrid materials for medical and biological applications.^7,8^ The dendrimers could stabilize the colloidal Au particles in aqueous solution at concentrations where unmodified colloids agglomerate. Additionally, the size of the colloids is a function of dendrimer generation.^8^ As a new class of highly branched, monodispersed, and synthetic macromolecules, poly amidoamine (PAMAM) dendrimers have attracted a great deal of interest in the development of various biological applications.^9-14^ The unique structural characteristics of PAMAM dendrimers allow one to design various dendrimer-metal nanocomposite.^14^ Recently, applicability of PAMAM to deliver anticancer drugs and other bioactives is well-reported in the literatures, especially dendrimers belonging to the fourth (G4) and fifth (G5) generation of PAMAM class.^7,10,15-17^ Poly amidoamine dendrimers contain a variety of functional groups capable of complexing metal cations, as well as interior voids in their structure capable of hosting small metal components and protecting them from further aggregation.^18^

The development of AuNP-based strategies for the eradication of cancer cells is important, because effective therapies are frequently not available for rapidly progressing cancers.^19^ So far, many of the studies on AuNPs suggest that cancer cells are especially vulnerable to these particles. Thus, AuNP-based treatment can destroy cancer cells, with minimal injury to healthy cells.^20^

Breast cancer, which is most resulted in metastasis, is the leading cause of cancer death in women with more than a million‏ newly diagnosed cases annually worldwide.^21^ 4T1 breast carcinoma is a highly malignant and poorly immunogenic marine tumor model that resembles advanced breast cancer in humans, and is refractory to most immune stimulation-based treatments.^19,22^

MCF7 is a breast cancer cell line isolated in 1970 from a 69-year-old Caucasian woman. MCF7 is the acronym of Michigan Cancer Foundation-7, referring to the institute in Detroit where the cell line was established in 1973 by Herbert Soule and co-workers.^23,24^ Prior to MCF7, it was not possible for cancer researchers to obtain a mammary cell line that was capable of living longer than a few months.

Cytotoxicity assay is an appropriate method for screening new drugs within a short time in order to determine cell killing property of these chemical compounds regardless the mechanism of cell death. Usually in oncology research and clinical practices, in vitro testing is preferred prior to in vivo studies. MTT assay has been described as rapid, simple and reproducible method, widely used in the screening of anticancer drugs and to measure the cytotoxic properties.^25^

In the current study, the preparation of ^197^Au/PAMAMG4 and ^198^Au/PAMAMG4 is described and the cytotoxic properties of them against the MCF7, 4T1 cancerous cells and C2C12 normal cells were evaluated using MTT assay.

## Materials and Methods

### Materials

Materials used in this study included methanolic solution of PAMAMG4 dendrimer and MMT (3- (4,5-dimethylthiazolyl-2)-2,5-diphenyltetrazolium bromide) obtained from (Sigma-Aldrich, USA). Hydrogen tetracholoroaurate (HAuCl_4_. 3H_2_O), sodium borohydride (NaBH_4_), sodium hydroxide, DMSO (dimethyl sulfoxide) and Whatman paper were purchased from (Merck. Germany). RPMI medium 1640, DMEM medium, fetal bovine serum (FBS), gentamicin, streptomycin, penicillin G, trypsin, EDTA were obtained from (Gibco, USA). MCF7 human breast cancer cells, 4T1 mice breast adenocarcinoma‏ cells, and C2C12 mice muscle normal cell were obtained from National Cell Bank of (Iran, Pasteur Institute, Tehran, Iran).

### Synthesis and characterization of ^197^Au/PAMAMG4 and ^198^Au/PAMAMG4 nanohybrids 

^197^Au/PAMAMG4 was prepared by two consecutive stages: First, the complex between dendrimer and the gold anions was formed, then the complexed ions were chemically reduced by a reducing agent (NaBH_4_).^26-28^ Firstly 0.5μmol dendrimer was added to 5 mL of water with vigorously stirring. Then 5 mL of an aqueous solution of 0.5 μmol HAuCl_4_ was added to the above solution. After stirring for further 45 min, procedure followed by addition of a basic aqueous solution of sodium borohydride (25 mM NaBH_4_ in 0.3 M NaOH). The prepared light yellow solution turned to brown, indicating the formation of colloidal gold. The stirring process was continued for 1 h to complete the reaction. The reaction mixture was then dialyzed against PBS (3 times, 4L) and water (6 times, 4 L) for 1 day to remove the excess reactants. At this stage ^197^Au/PAMAMG4 was prepared.

^197^Au captures neutrons very efficiently because of its large cross section. ^198^Au (t_1/2_=2.69 days) decays dominantly by beta-radiation (99%, 0.96 MeV) and with a small gamma component (0.98%, 1.1 MeV).^29^ Irradiation was performed at Tehran Research Reactor (TRR). Sample was irradiated in quartz vials for 2 hours, in core-face location, with a thermal neutron flux of 1 × 10^11^ncm^-2^s^-1^. The radionuclidic purity of the prepared ^198^Au/PAMAMG4 solution was determined for the presence of other radionuclides using purity germanium (HPGe) spectroscopy, through the detection of various interfering gamma emitting radionuclides. Final ^198^Au/PAMAMG4 solution (88 µCi, 50 µL) was incubated in the presence of freshly prepared human serum (300 µL) (Purchased from Iranian Blood Transfusion Organization, Tehran, Iran) at 37°C for 7 days. The stability study of ^198^Au/PAMAMG4 in final formulation was performing ITLC analysis using Whatman chromatography paper eluted with methanol: water (7:3) mixture. 

Spectroscopic analyses of the samples were performed using a Fourier transform infrared (FT-IR) spectrometer (Perkin–Elmer 843). Zeta-potential measurements were performed using (Malvern, U.K) with a red diode laser at 633 nm. Proton NMR spectra were conducted on a nuclear magnetic resonance spectrometer (Bruker Avance III 400MHz, Germany) using commercially available D_2_O as a solvent and Tetramethylsilane (TMS) as an internal standard. The morphology of prepared nanocomposites was studied by transmission electron microscopy (TEM, Philips-EM208S) and the content of Au in ^198^Au/PAMAMG4 was determined by means of energy dispersive X-ray spectrometer (EDX, Oxford INCA, UK). EDX analysis was performed using lyophilized nanocopmosite sample without gold coating.

### Cell culture and in vitro study 

The MCF7 human breast cancer‏ cells, 4T1 mice breast adenocarcinoma cells and C2C12 mice muscle normal cells were regularly cultured in RPMI 1640 medium and DMEM medium, respectively, supplemented with 10% FBS (Fetal bovine serum) and 1% penicillin-streptomycin at 37°C and 5% CO_2_. The cytotoxicity of the PAMAMG4, ^197^Au/PAMAMG4 and ^198^Au/PAMAMG4 were investigated by an MTT cell viability assay. MCF7, 4T1 and C2C12 cells were seeded onto a 96-well plate at a density of 4× 10^3^ cells per well and cultivated in 100 μL of their mentioned medium under 37°C and 5% CO_2_ for 24 h .^30^ After that, the mediums were replaced with fresh RPMI and DMEM containing PAMAMG4, ^197^Au/PAMAMG4 or ^198^Au/PAMAMG4 with different concentrations ranging from 50 nM to 400 nM and the cells were incubated under 37 °C and 5% CO_2_. The MTT assay was applied to study the cell viability after 24 h, 48 h and 72 h of drugstreatment. MTT (5.0 mg mL^−1^, 20 μL) was added into each sample and the cells were incubated at 37 °C and 5% CO_2_ for 4 h. Thereafter, the medium was removed and 150μL dimethylsulfoxide (DMSO) was added to dissolve the formazan crystals. The assays were performed according to the manufacturer’s instructions using a Biochrom Scientific Anthos 2020 ELISA reader (Biochrom Scientific, U.K) at 570 nm.^31,32^

All the experiments were repeated at least three times. Results are presented as the mean ±SD. Statistical comparisons were carried out using a three and four way analysis of variance ANOVA with post hoc testing for comparison of Sidak tests. Pair-wise comparisons between treatments were made using independent sample t-test (IBM SPSS Statistic 23, Microsoft Excel). A p-Value of < 0.05 was considered statistically significant.

The changes of 4T1 cell morphology after treatment with the ^197^Au/PAMAMG4 and ^198^Au/PAMAMG4 nanocomposites was studied by microscopic visualization. The morphology of the cells was observed using an invert optical microscope (Nikon, Tokyo, Japan) under a magnification of 200×.

## Results and Discussion

### Characterization of ^197^Au/PAMAMG4 and ^198^Au/PAMAMG4 nanocomposite

**Figure 1:**
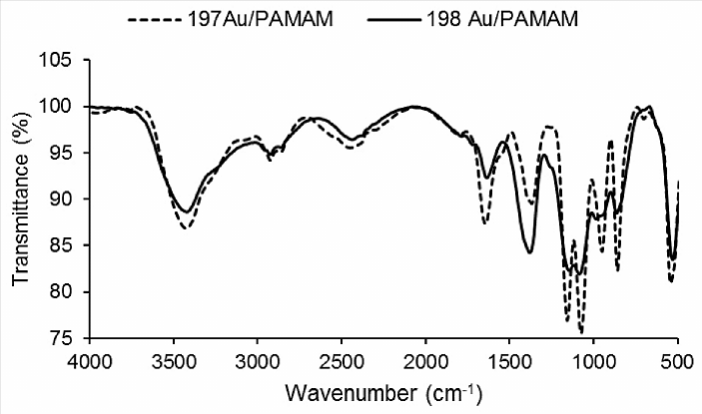
FT-IR spectrum of ^197^Au/PAMAMG4 (dash curve) and ^198^Au/PAMAMG4 (solid curve) nanocomposites.

Figure 1 shows a transmission infrared spectrum of ^197^Au/PAMAMG4 and ^198^Au/PAMAMG4 nanocomposites. The absorption peak at 3353cm^-1^ corresponded to N–H deformation vibration of tertiary amide, while the peak centered at 3287cm^-1^ was assigned to the N–H stretching vibration arising from its association. The next two peaks at 2944cm^-1^ and 2844cm^-1^ indicated unsymmetrical and symmetrical stretching vibration of methylene.^33^ The amide band present at 1640cm^-1^ is characteristic of the dendrimer branches.^8^ Also, N-H bending exhibits broad band at about 1640cm^-1^ for primary amine. The stretching vibration of C-N bonds is appeared at 1368cm^-1^. The two peaks at 1132cm^-1^ and 1202cm^-1^ were related to the stretching vibrations of tertiary amine and primary amine, respectively. N-H out of plane bending occurs at 800cm^-1^and the peak at 550cm^-1^ showed the bending vibration of tertiary amine.^34^ Comparison of FTIR curve of non-active ^197^Au/PAMAMG4 compound with radioactive ^198^Au/PAMAMG4 exhibited that the N-H band present at 1640cm^-1^ is stronger at non-active complex. Furthermore, the intensity of the peak assigned at 1368cm^-1^ is increased in the radioactive complex. It could be due to the cleavage of some N-H bands of primary amines and production of some new tertiary amines as a result of polymerization of nanocomposite particles under described neutron bombarding condition. Partial cross-linking of the organic component (radiation polymerization) was also reported by Khan and co-workers.^30^ Moreover, careful comparison of two curves reveals that neutron bombarding of ^197^Au/PAMAMG4 induced a small shift in the stretching vibration of C-N bond of prepared ^198^Au/PAMAMG4 to higher energy.

**Figure 2:**
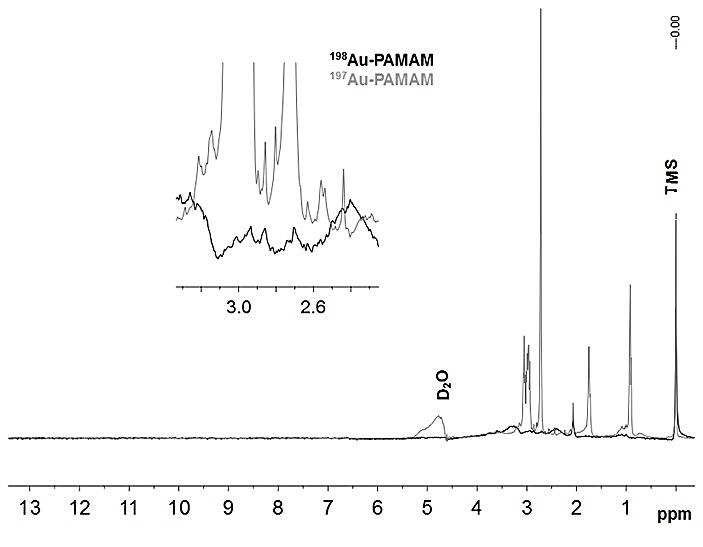
^1^H NMR spectra of ^197^Au/PAMAMG4 and ^198^Au/PAMAMG4 nanocomposites.

^1^H Nuclear Magnetic Resonance spectra of ^197^Au/PAMAMG4 and ^198^Au/PAMAMG4 nanocomposites are shown in Figure 2. Broadened peaks in the ^1^H NMR spectrum of ^198^Au/PAMAMG4 nanocomposite indicate partially of polimerization crosslinked PAMAMG4 during the neutron irradiation. The exact mechanism of polimerization is not known up to now. According to Tang and co-workers reports, the radical mechanisms and the local heat effects causing beta-alanine crosslinking, and in retro-Michael reaction may be responsible to take place a rearrangement of the molecular structure and finally polymerization.^35^

**Figure 3:**
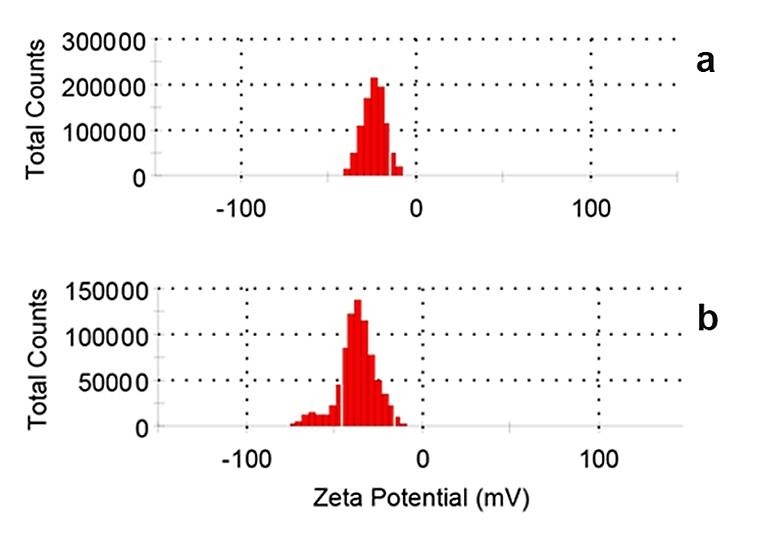
Zeta potential (ZP) of ^197^Au/PAMAMG4 and ^198^Au/PAMAMG4

Zeta-potential measurements for ^197^Au/PAMAMG4 and ^198^Au/PAMAMG4 were also carried out to investigate the surface potential changes after the neutron irradiation. As shown in Figure 3, the negative potential of the ^197^Au/PAMAMG4 (-23.9 mV) significantly changed after the neutron bombarding and producing of ^198^Au/PAMAMG4 (-37.6 mV). Decreasing of surface positive charge could be related to involvement of primary amine terminal groups in the ensuing polymerization reactions during neutron bombardment.^29,36^

**Figure 4:**
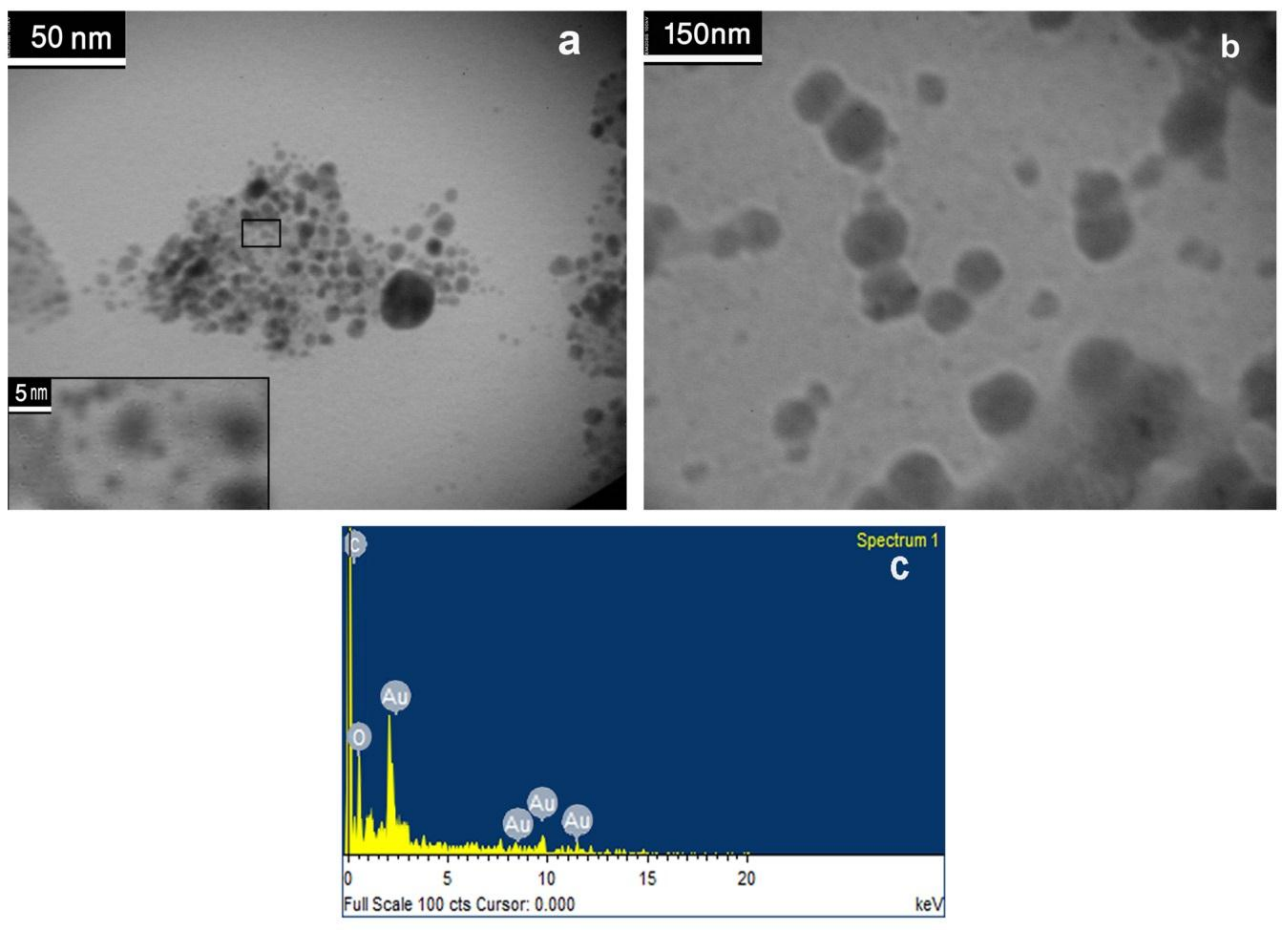
TEM images of ^197^Au/PAMAMG4 (a) and ^198^Au/PAMAMG4 nanocomposites(b), and EDX analysis of ^198^Au/PAMAMG4 (C).

Transmission electron microscopy analyses of nanoparticles were performed to understand the morphology and size of particles. TEM images (Figures 4a and b) show spherical shape for ^197^Au/PAMAMG4 and ^198^Au/PAMAMG4 compounds. Comparision of two pictures reveals that ^198^Au/PAMAMG4 has larger particle size compared to ^197^Au/PAMAMG4. This could be related to partial polymerization of PAMAMG4 due to neutron bombardment.^29^

**Figure 5:**
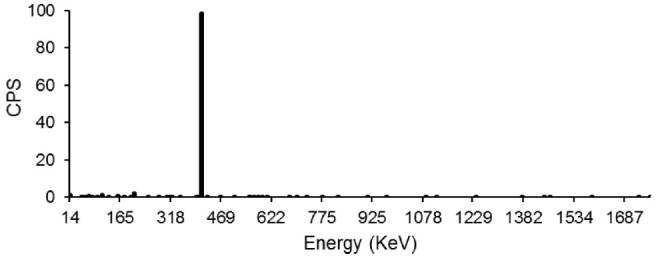
HPGe spectrum for 198Au/PAMAMG4 nanocomposite.

The radionuclidic purity of the ^198^Au/PAMAMG4 nanocomposie was checked by gamma-ray emission spectroscopy. Two main radioisotopes of gold have charming nuclear properties that make them desirable for imaging and therapy purposes. ^198^Au has a moderate-energy beta maximum (0.96 keV) making it a good candidate for therapy, and a gamma emission (411 keV) that allows for in vivo tracking and dosimetry calculation. These properties led to the medical use of ^198^Au as a brachytherapy agent for breast cancer. A second radioisotope, ^199^Au, has a low-energy beta maximum and emits a 158keV gamma that is easily detected by SPECT camera.^37^ Figure 5 shows the HPGe spectrum of the prepared compound. The prepared radioactive sample exhibited a characteristic gamma peak at 411KeV related to ^198^Au (t_1/2_ = 2.69 days), and there is no significant peak related to the ^199^Au isotope in the HPGe spectrum.

**Figure 6:**
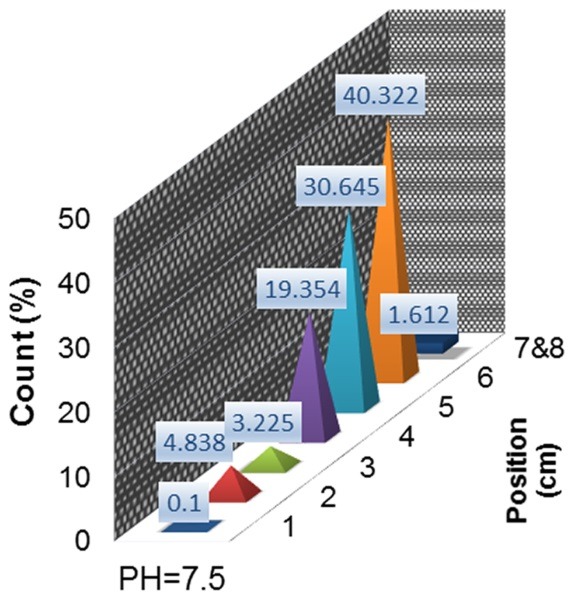
ITLC chromatogram of ^198^Au/PAMAMG4 at pH=7.5 with methanol: water (7:3) mixture and using Whatman 3 MM.

Figure 6 exhibits ITLC chromatogram of ^198^Au/PAMAMG4 at pH=7.5. The ^198^Au/PAMAMG4 complex in presence of human serum at 37 °C was found to be stable in final pharmaceutical sample and its radiochemical purity was calculated to be above 93.5% a week after the preparation by using Whatman 3 MM paper eluted with methanol: water (7:3) mixture.

### Cytotoxicity of ^197^Au/PAMAMG4 and ^198^Au/PAMAMG4 nanocomposites

**Figure 7:**
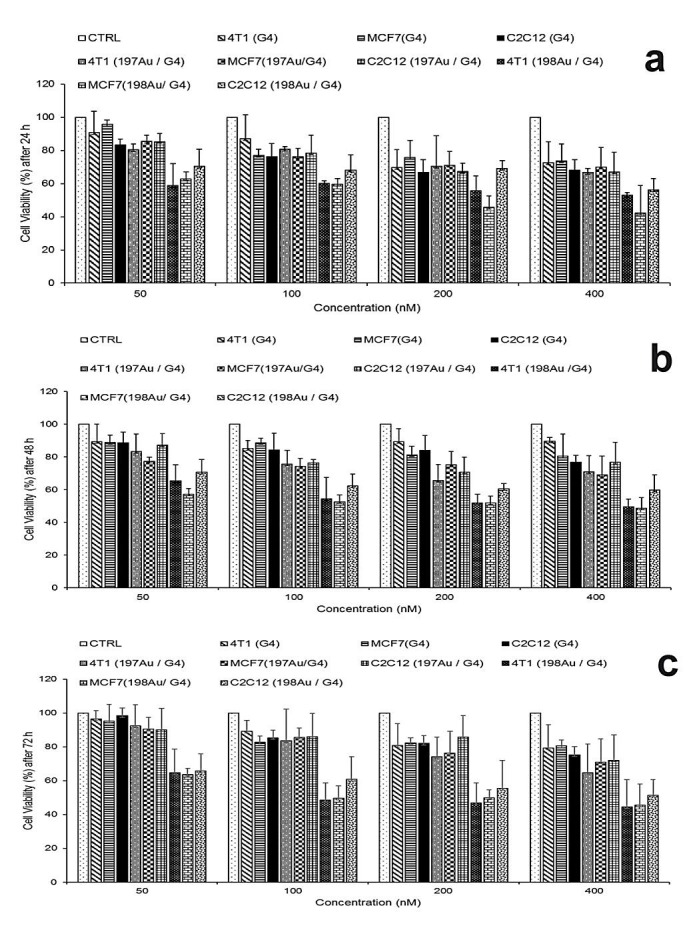
MTT-assay of the viability of MCF7, 4T1and C2C12 cells after treatment with different concentrations of PAMAMG4, ^197^Au/PAMAMG4 and radioactive ^198^Au/PAMAMG4 for 24, 48 and 72 h.

The cytotoxicity of the radioactive ^198^Au/PAMAMG4 and non- radioactive ^197^Au/PAMAMG4 against MCF7, 4T1 and C2C12 cells were assessed using the MTT assay. The MTT assay is based on the ability of a mitochondrial dehydrogenation enzyme in viable cells to cleave the tetrazolium rings of the pale yellow MTT and form formazan crystals with a purple color.^38^ Therefore; the number of surviving cells is directly proportionate to the level of the formed formazan. Figures 7a, b and c reveal the cell viability of the cultured MCF7, 4T1 and C2C12 cells treated by 50, 100, 200 and 400 nM‏ of PAMAMG4,^197^Au/PAMAMG4 and ^198^Au/PAMAMG4 solutions. Control groups use only medium.

Assessing the effect of drugs including PAMAMG4, ^197^Au/PAMAMG4 and ^198^Au/PAMAMG4 on viability of three cell lines of MCF7, 4T1 and C2C12 in various concentrations after 24h incubation, based on the results of three way ANOVA showed a three way interactions of cell lines* drugs *concentrations as (P<0.001).

The result of all two ways interactions are as follow: cell lines* drugs (P<0.001), cell lines*concentrations (P<0.001), and drugs *concentrations (P<0.001), and for all main effects of cell lines, drugs and concentrations (P<0.001) is obtained. This means that the effect of PAMAMG4, ^197^Au/PAMAMG4 and ^198^Au/PAMAMG4 on viability of each of the cell lines varies as a function of the concentration of drugs.

Additionally considering the significance of the three ways interaction, the results of Sidak simultaneous post hoc tests showed the lowest amount of viability in MCF7 and 4T1*^198^Au/PAMAMG4*concentration 400 (C400). In the other words, after 24 hours, treatment of MCF7 and 4T1 cell line with ^198^Au/PAMAMG4 leads to lowest amount of viability in the highest level of concentration (Figure 7a).

To investigate how drugs affect on the viability of MCF7, 4T1 and C212 cell lines in various concentrations after 48 hours incubation, the results of three way ANOVA showed significant three way interactions of cell lines* drugs*concentrations with (P < 0.001).

For all two ways interactions cell lines* drugs, cell lines*concentrations and drugs*concentrations P is lower than 0.001. Also for all main effects of cell lines, drugs and concentrations P are lower than 0.001.

Furthermore, taking into account the significance of the three ways interaction, the results of Sidak post hoc tests showed the significant lower values of viability for MCF7 and 4T1*^198^Au/PAMAMG4 *(C400,C200 and C100) and C2C12*^198^Au/PAMAMG4*(C50) levels. The other levels of three factors were significantly in higher amounts of viability. Hence, after 48 hours, ^198^Au/PAMAMG4 of MCF7 and 4T1 cell lines lead in lower amount of viability in the higher level of concentration (Figure 7b).

After 72 hours incubation, the results of three way ANOVA to study of the effect of drugs materials on viability of three cell lines of MCF7, 4T1 and C2C12 in various concentrations of drugs showed significant three ways interactions of cell lines* drugs *concentrations (P < 0.001), all two ways interactions of cell lines* drugs (P < 0.001), cell lines*concentrations (P < 0.001), and drugs *concentrations (P < 0.001) as well as for all main effects of cell lines, drugs and concentrations (P < 0.001). In the other words, the effects of drugs including PAMAMG4, ^197^Au/PAMAMG4 and ^198^Au/PAMAMG4 materials on the cell viability vary as a function of the cell lines for each concentration. Furthermore, pertaining to the significance of three ways interaction, based on the results of Sidak post hoc tests, lower values of viability were observed in MCF7 and 4T1*^198^Au/PAMAMG4 *(C400 to 50 nM) levels, and other levels of the three factors had significantly higher amount of viability. As shown in Figure 7c, after 72 hours, the effect of ^198^Au/PAMAMG4 towards 4T1 cell line results in the lowest amount of viability in higher level of concentration. To compare the changes in the viability during three time points of 24, 48 and 72 hours along with the effect of cell lines, drugs and concentrations, the findings of four way analysis of variance revealed the significant four ways interaction effect of times*cell lines* drugs*concentrations (F(24,126)=7.42, P < 0.001). It means that the changes of cell viability during three time points of 24, 48 and 72 hours varies for cell lines, each drug (PAMAMG4, ^197^Au/PAMAMG4, and ^198^Au/PAMAMG4) and within the levels of concentrations (C50 to C400). As can be seen the effect of cell lines, drugs (PAMAMG4, ^197^Au/PAMAMG4 and ^198^Au/PAMAMG4) and concentrations leads in significantly the lowest amount of viability after 72 hours based on the results obtained from the Sidak post hoc tests (P < 0.05).

The cytotoxicity effects of the compounds towards the 4T1 cell line were further confirmed by microscopic visualization of changes of the cell morphology after treatment with the ^197^Au/PAMAMG4 and ^198^Au/PAMAMG4. It is reported that the treatment of cells with some test compounds induced morphological alterations such as retraction of cytoplasmatic expansions; detachment, formation of the round shaped cells as well as membrane blebs, all suggestive the induction of programmed cell death.^39^

**Figure 8:**
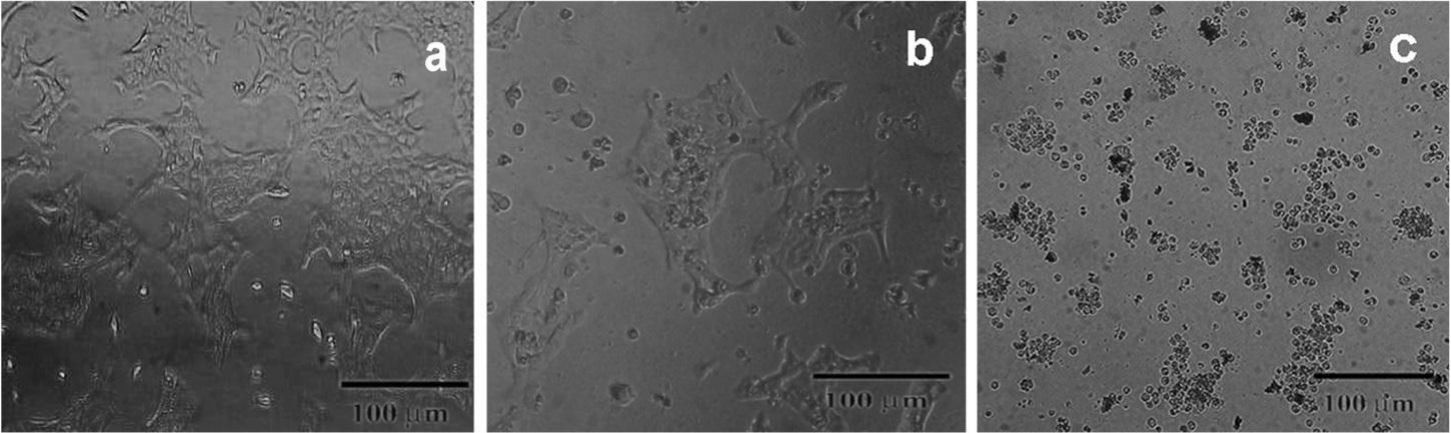
photomicrographs of the 4T1 cells (inverted microscopy, ×200)‏ without treatment (a), and the 4T1 cells treated with ^197^Au/PAMAMG4 (b) and ^198^Au/PAMAMG4 (c).

Figure 8a shows the photomicrographs of the 4T1 cells, and Figures 8b and 8c reveal the photomicrographs of the 4T1 cells treated with ^197^Au/PAMAMG4 and ^198^Au/PAMAMG4, after 24 h, respectively. As shown in Figure 8b, minor morphological alterations are shown in the 4T1 cells treated with ^197^Au/PAMAMG4 nanocomposite with concentration (400 nM) compared with the untreated cells. According to Figure 8b, the rounded and detached cells can be visualized in the cells treated with ^197^Au/PAMAMG4 to some extent. In contrast, Figure 8c shows that a significant portion of the cells became rounded and non-adherent as a result of ^198^Au/PAMAMG4 treatment. This phenomenon could be related to apoptosis of 4T1 cells due to ^198^Au/PAMAMG4 (16.64 μCi) treatments. These results are consistent with the MTT assay data.

## Conclusion

In this study, radioactive ^198^Au/PAMAMG4 nanocomposite was prepared successfully by neutron bombardment of synthesized non-radioactive ^197^Au/PAMAMG4. ^1^H NMR, FT-IR and TEM analyses reveals partially cross-linked polymerization of PAMAMG4 in the repared neutron bombarded sample (^198^Au/PAMAMG4). In vitro anti-cancer assessments of radioactive ^198^Au/PAMAMG4 mater and non-radioactive ^197^Au/PAMAMG4 with regard to the MCF7, 4T1 and C2C12 cell lines were investigated. Analysis of variance followed by Sidak post-hoc test, shows that the toxicity of ^198^Au/PAMAMG4 is significantly deferent from ^197^Au/PAMAMG4 and PAMAMG4 on all of the three cell lines. The toxicity of ^198^Au/PAMAMG4 is more on cancerous cells compared to normal cells especially in higher level of concentrations after 24, 48 and 72 hours (P<0.05). In conclusion, ^198^Au/PAMAMG4 inhibited growth of MCF7 and 4T1 breast cancer cells in a dose- and time-dependent manner *in vitro.* Moreover, the effect of cell lines, drug types and concentrations of drugs leads to the lowest amount of viability after 72 h compared with 24 h and 48 h.

## Acknowledgments

Support by Nuclear Science and Technology Research Institute of Atomic Energy Organization of Iran and Iran National Science Foundation (INSF) is greatly appreciated.

## Ethical Issues

Not applicable.

## Conflict of Interest

The authors declare no conflict of interests.
